# The Effect of Physiotherapy on Arthrogenic Muscle Inhibition After ACL Injury or Reconstruction: A Systematic Review

**DOI:** 10.3390/life14121586

**Published:** 2024-12-02

**Authors:** Maria Paço, Maxence Peysson, Elona Dumont, Mário Correia, Anna Quialheiro, Paula Chaves

**Affiliations:** 1 CESPU, Instituto Politécnico de Saúde do Norte, Escola Superior de Saúde do Vale do Ave, 4760-409 Vila Nova de Famalicão, Portugal; maria.paco@ipsn.cespu.pt (M.P.); mario.correia@ipsn.cespu.pt (M.C.); paula.chaves@ipsn.cespu.pt (P.C.); 2 H2M—Health and Human Movement Unit, Polytechnic University of Health, CESPU, CRL, 4760-409 Vila Nova de Famalicão, Portugal; 3 UNIPRO—Oral Pathology and Rehabilitation Research Unit, University Institute of Health Sciences (IUCS-CESPU), 4585-116 Gandra, Portugal; 4 IA&Saúde—The Artificial Intelligence and Health Research Unit, Polytechnic University of Health, CESPU, CRL, 4760-409 Vila Nova de Famalicão, Portugal

**Keywords:** neural inhibition, AMI, quadriceps, physiotherapeutic intervention, muscle rehabilitation

## Abstract

Arthrogenic muscle inhibition (AMI) following ACL injury or reconstruction is a common issue that affects muscle activation and functional recovery. Thus, the objective of this study was to systematize the literature on the effects of physiotherapy interventions in the rehabilitation of AMI after ACL injury or reconstruction. A systematic review was conducted following the PRISMA guidelines. The risk of bias was evaluated using the PEDro scale and the Cochrane risk of bias tool. Searches were performed in the PubMed, Google Scholar, Cochrane Library, and EMBASE databases. Randomized controlled trials involving patients with ACL injuries or ACL reconstruction were included. Twenty studies were included. Fifteen evaluated the effects of exercise, showing significant improvement. Seven studies examined electrotherapy, with neuromuscular electrical stimulation and high-frequency therapy combined with exercise showing improvements in muscle strength, pain, and joint range of motion. Nine studies explored interventions like motor imagery, cryotherapy, taping, and vibration. When performed before exercise, motor imagery and cryotherapy improved cortical activity and muscle recovery. Kinesio taping reduced edema and pain better than exercise alone. Vibration showed inconsistent results across three studies. Methodological quality varied between 5 and 8 on the PEDro scale, with moderate-to-low risk of bias. Structured exercise should be the first-line intervention, but combining it with other therapies enhances rehabilitation. The study protocol was registered in the PROSPERO database (CRD42023425510).

## 1. Introduction

Anterior cruciate ligament (ACL) injuries are among the most common and debilitating sports-related injuries, often leading to long-term functional impairments if not properly treated. A key challenge in post-injury rehabilitation is overcoming arthrogenic muscle inhibition (AMI), a process where neural inhibition prevents the adequate activation of the quadriceps muscle. This inhibition can lead to muscle atrophy, particularly in the vastus medialis, extension deficits, persistent knee pain, and joint instability [1,2]. AMI is frequently observed following ACL injuries and during rehabilitation after ACL reconstruction, with an incidence of up to 56% six weeks post injury [3]. Thus, addressing AMI is critical, as failure to do so can result in suboptimal recovery and long-term disability.

From a pathophysiological perspective, AMI affects not only the local joint—due to mechanisms such as inflammation, joint effusion, and altered articular receptor sensitivity—but also the peripheral nervous system, through increased inhibitory interneuron activity and gamma loop dysfunction. Moreover, it impacts the supraspinal level, manifesting as decreased intracortical inhibition and reduced frontal cortex activity [4]. It is described that peripheral mechanisms can alter afferent input to the central nervous system, leading to changes in motor neuron excitability and cortical inhibition. These alterations disrupt the normal neural signaling between joint receptors and the central nervous system, perpetuating muscle inhibition and further exacerbating the severity of AMI [1,2,3]. Understanding this interaction is crucial for developing effective rehabilitation strategies.

AMI can be categorized into different degrees, ranging from mild cases without extension deficits (grade 1) to severe cases requiring prolonged rehabilitation (grade 2b) and even chronic irreducible extension deficits (grade 3) [3]. Current therapeutic interventions aim to counteract these inhibitory processes by targeting both peripheral and central mechanisms [5,6,7,8,9,10]. Various physiotherapy interventions have been described, including structured exercise, neuromuscular electrical stimulation (NMES), motor imagery, blood flow restriction therapy, and cryotherapy [11,12]. Despite this, there is ongoing debate regarding the role of adjunctive therapies, particularly concerning their efficacy, optimal timing, and potential risks. Some studies suggest benefits in early intervention, while others do not support this [3,4,5,6,7,8,9,10], indicating a need for further research to establish clear guidelines. Having said this, structured exercise remains central to the rehabilitation process, and open kinetic chain exercises have been shown to pose no risk in the immediate postoperative period, allowing rehabilitation to begin as early as the day after surgery [13,14,15]. Furthermore, combining structured exercise with other modalities, including electrotherapy, cryotherapy, and vibration, has shown the potential to enhance motor recovery [11,12,14,16].

However, persistent quadriceps motor deficits have been observed up to 12 months post ACL reconstruction, indicating the need for a more integrative approach to treatment [17]. There remains limited consensus on the most effective combination of therapies to address both the central and peripheral components of AMI, with conflicting results across studies. Moreover, research has not demonstrated significant differences between eccentric, concentric, or isometric contractions in terms of their effectiveness for AMI rehabilitation [18,19,20].

Thus, this systematic review aims to synthesize the available literature on the effects of physiotherapy interventions in the rehabilitation of AMI following ACL injury or reconstruction. By addressing both peripheral and central mechanisms, this review seeks to clarify the most effective therapeutic combinations and offer guidelines for clinical practice.

## 2. Materials and Methods

This systematic review was conducted following the PRISMA guidelines. The study protocol was registered in the PROSPERO database (CRD42023425510).

The following review question was considered: What are the effects of non-invasive physiotherapy interventions on AMI in individuals with anterior cruciate ligament (ACL) injury or post-reconstruction surgery?

### 2.1. Eligibility Criteria

All inclusion and exclusion criteria for the studies were established a priori, defined with the PICOS acronym (Population, Intervention, Comparison, Outcomes, Study Design):

Population: Participants with a history of ACL injury (with or without arthroplasty) less than one year ago.

Intervention: All studies evaluating the effect of non-invasive physiotherapy techniques were included.

Comparison: Other interventions or a control/sham group.

Outcomes: AMI indicators, such as knee extension range, quadriceps strength, and electromyographic activity of the quadriceps, and other muscle-function-related indicators, such as functionality, balance, proprioception, pain, joint effusion, muscle histological structure, and central activation ratio.

Study Design: Randomized controlled trials.

No date or language limits were set for article selection, and exclusion criteria included the presence of other knee pathologies (meniscal injuries, patellofemoral instabilities, arthrofibrosis, fractures, multi-ligament injuries, neurovascular damage, or others), injury to the contralateral knee, presence of cognitive alterations, and studies with AMI induced by intra-articular fluid injection.

### 2.2. Information Sources and Search Methodology

The following databases were used: PubMed, Google Scholar, The Cochrane Library, and Embase. The search period was from 20 November 2022 to 7 February 2023. To create search expressions in different databases, terms such as quadriceps inhibition, arthrogenic muscle inhibition, quadriceps weakness, anterior cruciate ligament (ACL), physiotherapy, physical therapy, arthrofibrosis, and bilateral impairment were used. These terms were combined using the Boolean operators AND, OR, and NOT. The specific search strategies and their results used in different databases are in Appendix A. The articles were saved in the EndNote Library, and duplicate articles were excluded.

### 2.3. Study Selection

**Figure 1:**
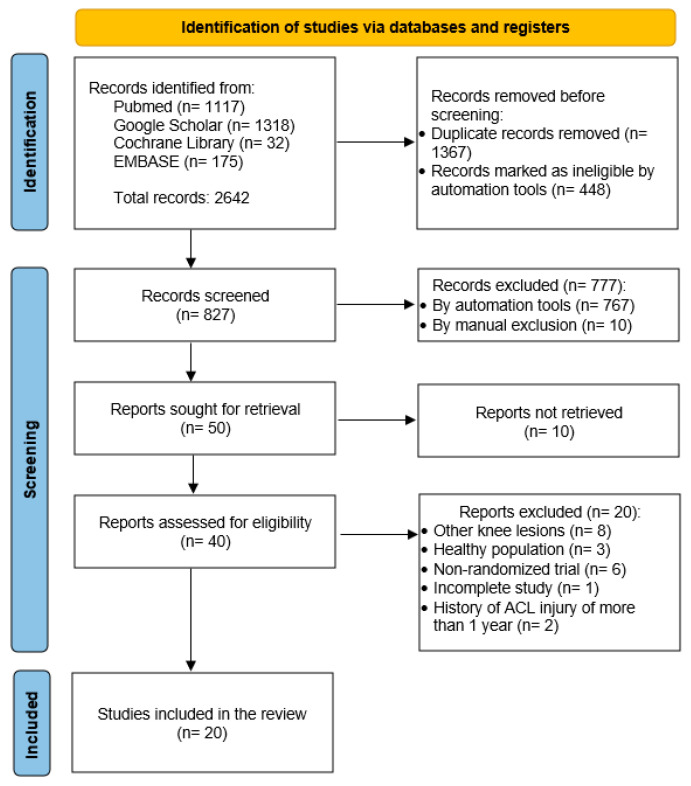
PRISMA flow diagram.

In the first phase, the titles and abstracts of the studies resulting from the search in the different databases were analyzed. Subsequently, the selected articles were read in full by two independent researchers to verify the defined eligibility criteria (Figure 1). In case of disagreement, a third researcher was consulted.

Data from the included articles were then extracted, collecting the following information: authors; year; population; intervention; outcome; measurement instruments; results; and conclusion.

### 2.4. Methodological Quality Assessment

The methodological quality of the 20 included articles was assessed independently by two researchers using the PEDro scale and the Cochrane risk of bias tool. Disagreements were resolved by consulting a third researcher.

Regarding the PEDro scale, a score between 0–3 is considered “poor”, 4–5 “fair”, 6–8 “good”, and 9–10 “excellent”. A score between 8–10 is considered “optimal” for studies evaluating complex interventions (such as exercise), though these classifications are not yet validated but are recommended [21].

## 3. Results

The initial search yielded 2642 articles, to which the previously described selection criteria were applied, resulting in a total of 20 studies (Figure 1). The publication date of the included studies ranged from 1991 to 2022. Table 1 summarizes the characteristics of the studies, the results, and the PEDro score.

### 3.1. Therapeutic Exercise

Out of the 20 articles included in this systematic review, 15 assessed the effects of therapeutic exercise, in various forms, on AMI [22,25,27,28,29,30,31,32,33,34,35,36,37,39,40], which are described below.

#### 3.1.1. Structured Exercises

Among the selected studies, 15 utilized structured exercises [22,25,27,28,29,30,31,32,33,34,35,36,37,39,40]. Exercise loads varied across studies: six studies adapted the number of sets, repetitions, or workloads according to the patient’s condition [22,28,29,31,37,40], while others used uniform loads for all participants [27,33,39]. In some studies, the load was determined by the participant [32] or not reported at all [25,34,35,36].

Four studies also included a home-based structured exercise program [22,29,30,31].

Regarding outcomes, 11 studies measured the impact of structured exercises on muscle strength, with statistically significant improvements in strength (*p* < 0.05) [22,25,27,28,29,30,31,32,35,37,40]. Various methods were used to evaluate strength, including maximum voluntary isometric contraction (MVIC) [22,29,30,31,35,37,40] and maximum concentric contraction [25,27,28]. One study utilized the 10RM test [32].

Range of motion was assessed in three studies [22,32,37], with two showing significant improvements in knee extension (*p* < 0.05) [22,37]. Structured exercises also positively affected cortical activity [30,39], post-operative muscle atrophy [33], pain reduction [22,32,36,37], proprioception [28,34], and functionality [27,29,30,32,37].

#### 3.1.2. Blood Flow Restriction (BFR)

Two studies assessed the effects of BFR therapy [32,33], which uses an inflatable cuff to restrict blood flow during low-intensity resistance training and is thought to produce the same muscular adaptations as high-intensity resistance training [42,43]. However, only one of these studies [32] reported significant reductions in pain and edema, though the results were not statistically significant compared to the control group.

#### 3.1.3. Cross-Exercises

Two studies investigated cross-exercises [29,40], with both showing significant improvements in muscle strength during the early phases of treatment (*p* < 0.05) [29,40] but no significant differences in functional improvements (*p* > 0.05) [29].

#### 3.1.4. Biofeedback

Three studies examined the effect of biofeedback on AMI rehabilitation [24,26,34]. While biofeedback led to faster improvements in knee extension [24] and muscle strength [24,26], it was not superior to other interventions regarding proprioception and functional balance [34].

### 3.2. Electrotherapy

Seven articles in this systematic review assessed the effect of electrotherapy, in various forms, on AMI [26,27,30,34,35,37,41].

#### 3.2.1. Neuromuscular Electrical Stimulation (NMES)

NMES facilitates muscle and nerve stimulation using electrodes placed on the skin over the desired area, enhanced by an electrical current passing between the anode and cathode. NMES can improve motor recovery following denervation by stimulating motor units [44,45]. Three studies assessed the effect of NMES combined with exercise compared to exercise alone [27,35], or exercise + biofeedback [26], focusing on muscle strength in patients undergoing ACL reconstruction. A total of 160 patients were included in these studies, with one study showing a statistically significant improvement in muscle strength and knee function when compared to exercise alone (*p* < 0.001) [27].

#### 3.2.2. Transcutaneous Electrical Nerve Stimulation (TENS)

TENS is another form of electrotherapy with some controversy surrounding its effectiveness [16,46]. TENS works by placing electrodes near the painful area to reduce pain. Its analgesic effect is thought to be based on the gate control theory and the release of endogenous opioids [47,48,49]. Only one randomized controlled trial was included in this review, and it found no significant differences in muscle strength, functionality, or the central activation ratio (*p* = 0.94). Additionally, TENS did not significantly reduce pain compared to the other groups (*p* > 0.05) [30].

#### 3.2.3. Transcranial Direct-Current Stimulation (tDCS)

tDCS is another type of electrical stimulation therapy that increases cortical excitability by applying electrodes on the scalp. The method has been researched for its effects on neurological diseases and musculoskeletal pathologies, such as ACL injuries [50]. Two articles were included in this review [34,41]. In one study, after 10 sessions over four weeks, tDCS showed a statistically significant increase in proprioception and functional balance (*p* < 0.05), however without statistically significant differences when compared with exercise alone or exercise + biofeedback [34]. In another study, no significant effects were observed on muscle strength or function after a single session of tDCS compared to a sham intervention [41].

#### 3.2.4. High-Tone Therapy

High-tone therapy is a systemic electrostimulation method aimed at improving tissue metabolism and reducing pain, unlike NMES, which targets muscles and nerves. One study examined the effects of high-tone therapy in conjunction with a standard rehabilitation protocol on muscle strength, knee extension, function, and pain [37]. The study found statistically significant improvements in quadriceps strength (*p* < 0.05), knee extension (*p* < 0.001), and knee function (*p* < 0.05) compared to the control group, although there were no significant differences in pain reduction between groups.

### 3.3. Other Interventions

#### 3.3.1. Cryotherapy

Cryotherapy involves applying a medium or substance to the body that can lower the temperature of adjacent tissues. Two studies conducted by Hart et al. [30,31] compared the effects of cryotherapy alone, combined with exercises, exercises alone, and exercises combined with TENS, on muscle strength [30,31], the central activation ratio [30,31], and knee function, pain, extension, range of motion, and edema [30]. In one study, although there was a significant improvement in quadriceps strength in the group that had cryotherapy combined with exercise, no significant effect of cryotherapy was found when compared with the other groups (*p* > 0.05) [31]. Another study [30] reported a statistically significant improvement in muscle strength, function, and pain across all groups, with the comparison between groups showing no significant differences (*p* > 0.05). This study also showed that combining cryotherapy with exercise did not improve knee extension, range of motion, or edema (*p* > 0.05) [30].

#### 3.3.2. Motor Imagery

Motor imagery is a mental technique widely used by high-level athletes, where individuals visualize a specific movement (motor gesture) to activate the corresponding cortical areas, similar to performing the actual movement [51,52]. This technique can be applied in rehabilitation to facilitate the recovery of motor function, including in musculoskeletal injuries [53,54]. Only one of the included studies in this review used this technique [23]. The study compared the effect of a single motor imagery session combined with an exercise session to a control group that received the same interventions but with a sham passive mobilization session instead of motor imagery. Both groups showed significant improvements in motor threshold activation (*p* < 0.05). The motor imagery group achieved slightly better results than the control group at 80% to 150% of the motor activation threshold. The most significant result was observed at 150%, where the motor imagery group showed a +4.77 ± 3.1% increase in motor activation compared to a −0.004 ± 0.1% decrease in the control group [50].

#### 3.3.3. Kinesio Taping (KT)

Three studies assessed the effects of KT combined with standard rehabilitation (pain control, range of motion, proprioception, and muscle strength) on various outcomes, including pain, range of motion, edema, muscle activation, strength, and functionality in patients undergoing ACL reconstruction (ACLR) [22,36,38]. The studies concluded that postoperative KT application did not improve muscle strength [22,38], range of motion [22,36], function [36], or postural balance [38]. One study [36] evaluated KT’s effects on edema and reported a significant reduction in edema (*p* < 0.05). Two studies evaluated the effects of KT on pain [22,36], and only one found a greater reduction in pain in the group that received KT (*p* < 0.05) [22].

#### 3.3.4. Vibration

Local vibration or whole-body vibration therapy is a neuromuscular technique that induces oscillations producing rapid, short-duration changes in the length of the musculo-tendinous complex. Vibration therapy is thought to improve proprioception, balance, muscle trophism, and muscle strength when combined with exercise [55]. Three randomized controlled trials included in this review assessed the effects of vibration combined with exercise in post-ACLR patients [25,28,39]. All three evaluated quadriceps function (peak torque, central activation ratio, EMG activity), but only one found significant differences in favor of vibration (*p* < 0.05) [39]. One study [28] observed an improvement in postural control (*p* < 0.05) as well as a statistically significant improvement in knee function (*p* < 0.05).

### 3.4. Methodological Quality and Risk of Bias

The methodological quality of the included studies was assessed using the PEDro scale. As shown in Table 2, all studies demonstrated good methodological quality, except for two [35,38], which were classified as fair quality. Four studies that evaluated complex interventions were considered to have optimal methodological quality (score ≥ 8) [22,27,28,36].

**Figure 2:**
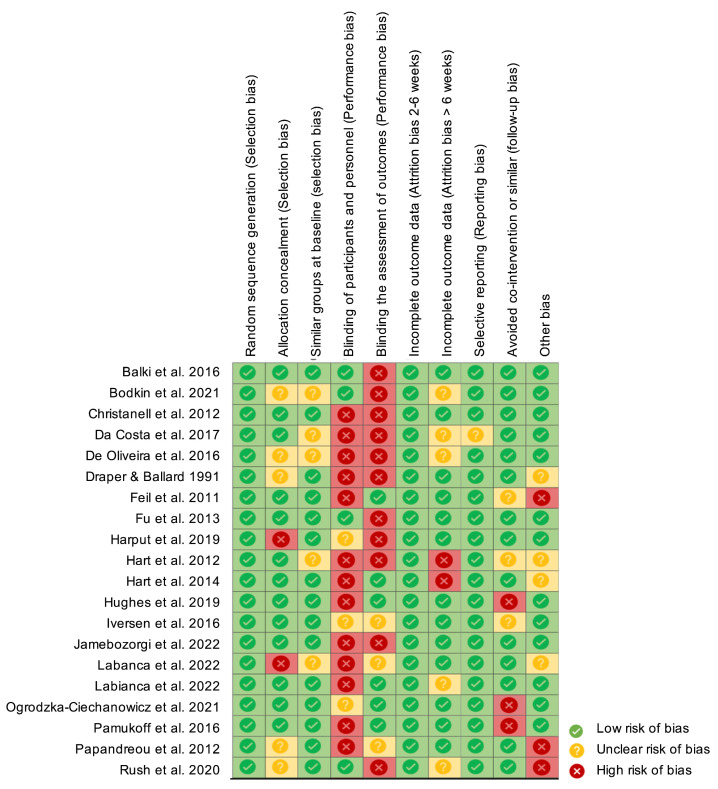
Cochrane risk of bias tool (Rob2) [22,23,24,25,26,27,28,29,30,31,32,33,34,35,36,37,38,39,40,41].

Figure 2 presents the risk of bias identified with the Cochrane risk of bias tool, showing a moderate-to-low risk of bias across the studies.

## 4. Discussion

This systematic review underscores the importance of structured exercise as the cornerstone intervention for addressing AMI following ACL injury or reconstruction. However, it is evident from the included studies that combining exercise with adjunctive therapies, such as NMES, cryotherapy, motor imagery, and other non-invasive interventions, results in superior outcomes compared to exercise alone. The heterogeneity in methodologies and interventions across the studies presents a challenge in drawing uniform conclusions. However, multimodal rehabilitation strategies appear more effective than single-modality approaches as they target both peripheral and central mechanisms of neural inhibition [3,4,9].

### 4.1. Therapeutic Exercises

#### 4.1.1. Structured Exercises

Structured exercise encompasses systematically designed physical activities aimed at improving specific aspects of physical fitness and function. It is essential in the rehabilitation of MIA and cruciate ligament injuries in general. This approach is described in many guidelines [14,56], and it is recommended that it should be planned and implemented as early as possible. Many studies have tried to determine the ideal modalities, and it seems that preoperative rehabilitation with a load of 50 to 90 percent of 1RM improves function and strength and reduces pain at 12 weeks after surgery compared to someone who has not undergone preoperative rehabilitation [57,58]. Open-kinetic-chain exercises seem to present no risk in the postoperative period, and it is even recommended that these, as well as isometrics in overcoming [13,59,60], are the two most suitable types of exercise in the acute phase. In the subacute and chronic phase, no modality, whether concentric, eccentric, or isometric, seems to be more effective than the others [14,18]. Therefore, it is recommended to plan, quantify, and alternate the contraction modalities according to the patient’s practice to be progressive and functional and allow a return to sports practice, with a low risk of recurrence. In addition to the peripheral effect, exercise also seems to have a very positive central effect on neuroplasticity and the excitability of motor areas [61,62,63].

Of the 15 articles selected, only 4 articles [28,29,31,40] used an adapted volume and workload to personalize the treatment better. The other articles used fixed sets and repetitions, with constant loads, which results in a constant volume and does not respect the principle of progressivity, which is one of the key principles of rehabilitation [64,65]. Only four studies implemented a home exercise protocol in addition to supervised sessions [22,29,30,31], which are known to be fundamental in the treatment of patients who have suffered an ACL rupture or reconstruction [66]. Despite protocols considered standard or questionable exercise choices, in all studies, improvements in strength, range of motion, functionality, proprioception, cortical activation, and muscle activation were observed with exercise.

This study confirms that exercise plays a central role in modulating MIA. However, only one study [31] investigated the effect of structured exercise on patients that had evidence of chronic quadriceps dysfunction (i.e., a quadriceps CAR of 90% or less) and that was considered AMI, since in 2014, no classification had been published yet. Future studies should focus on the effects of a rehabilitation program based on relevant, personalized, and progressive exercises in a population diagnosed with AMI according to the Sonnery-Cottet et al. classification [3].

From a clinical perspective, the reader should be aware that implementing tailored exercise regimens based on individual assessments can optimize rehabilitation outcomes. Regular monitoring and progression of exercises are essential to meet the evolving needs of patients.

#### 4.1.2. BFR

BFR involves placing an inflatable cuff or tourniquet on the proximal end of the limb to decrease blood flow during exercise. The interest would be that with low-intensity resistance training, the same muscular adaptations could be achieved as with high-intensity resistance training [11,12,13]. The physiological mechanism is not yet clear, and it is thought that intramuscular hypoxia may be responsible for vascular endothelial growth and elevated levels of metabolic stress accountable for hypertrophy [12,14,15].

The results did not show better results than the control group but equal results with a (theoretically) lighter load, which can be explained by the greater metabolic stress due to blood flow restriction, which is confirmed by other studies on this topic [32,67]. It could therefore be an interesting therapeutic tool in the acute phase, or in frail individuals, where the use of heavy external exercise loads may be contraindicative, but further research is still needed.

From a clinical perspective, clinicians should ensure the proper application of BFR, including appropriate cuff placement and pressure settings, to maximize benefits and minimize risks. Training in the application of BFR is also recommended.

#### 4.1.3. Cross-Over Exercises

The practice of cross-over exercises, also known as cross-exercise, involves performing unilateral exercises with the non-operated limb, aiming to induce adaptations in the operated limb [16,17,18]. Changes in corticospinal excitability of the trained limb, the decrease in corticospinal and inter-hemispheric inhibition seem to be responsible for these cross adaptations [16,18].

Future studies should investigate the dose and frequency to optimize the integration of cross-training exercises in the acute phase of rehabilitation protocols. According to the authors’ findings, cross-over exercises appear to be beneficial in limiting muscle atrophy but do not improve supra-spinal activity. There is no difference between concentric and eccentric exercises, and neither of the two contraction modalities allows for more adaptations than the other. Future studies in patients diagnosed with AMI grade Ia or higher should be conducted to confirm the usefulness of cross-training exercises in the case of post-ACL/ACLR rehabilitation in the presence of AMI.

For the clinician, incorporating cross-education exercises can be particularly useful in early rehabilitation stages when direct training of the injured limb is not feasible.

#### 4.1.4. Biofeedback

Biofeedback, or biological feedback, refers to a process that allows an individual to learn and control their physiological activity using electrodes that utilize bioelectricity or through simpler and less costly methods, such as motion guidance or goal-oriented exercise (reaching objects, timed duration, score, …), which allows for modulating cortical activity [19].

The three studies seem to confirm the relevance of using biofeedback in the acute phase of a knee extension deficit, proving to be more effective than electrostimulation or simple exercise [24,26,34]. The feedback of the strength developed makes it possible to improve cortical activity in the areas corresponding to the movements, thus reducing the neural inhibition of the quadriceps. However, none of these three trials measured the central activation ratio. As biofeedback is a process that allows for cortical activity to be modulated, it would also have been interesting to see if this was reflected in quadriceps activity and proprioception in the treatment of ACL injury.

In clinical practice, this therapeutic intervention can be easily integrated into the early stages of rehabilitation and opens playful possibilities once it can enhance patient engagement and adherence to rehabilitation protocols by providing immediate feedback on performance

### 4.2. Electrotherapy

#### 4.2.1. NMES

NMES facilitates the stimulation of muscles and nerves using electrodes placed on the skin over the desired area, aided by an electrical current passing from anode to cathode. Neuromuscular electrical stimulation can enhance motor recovery after denervation by stimulating motor units [44,45].

Two of the three included studies reported that NMES significantly improved patients’ muscle strength [27,35], probably because it allows for increasing the dose of stimulation during exercise. Again, the experimental and control groups did not receive the same amount of stimulation, because the addition of NMES increased the proportion of stimulation in the experimental groups, resulting in a higher volume or intensity. Future studies that take this into account should compare the two groups at the same volume or intensity to evaluate the effects of NMES compared to exercise.

Nonetheless, NMES seems to be a good way to improve motor unit excitability, restore normal quadriceps activity more quickly, and increase strength and function, even after several months. Thus, from a clinical perspective, incorporating NMES into rehabilitation can be particularly beneficial in the early postoperative period to maintain muscle mass and facilitate neuromuscular re-education.

#### 4.2.2. TENS

TENS is another type of electrotherapy that presents controversial results [32,33]. It involves placing electrodes near the painful area to decrease pain [34,40]. The analgesic effect is based on two principles of action: inhibition of nociceptive transmission (gate control theory) and release of endorphins (endogenous opioid substances) [29,40]. Further studies are needed to confirm the results, but so far, the use of TENS does not seem to be relevant in this clinical setting. Nonetheless, applying TENS prior to or during therapy sessions can reduce pain levels, possibly allowing for more effective engagement in rehabilitation activities.

#### 4.2.3. tDCS

tDCS is a form of electrical stimulation therapy where electrodes are placed on the patient’s head via a helmet. It is considered a non-invasive therapy that applies a low-intensity electrical current to the scalp, with controversial outcomes, potentially acting to increase activity and cortical excitability [27,30,31]. This approach has been explored in various neurological and psychiatric conditions, including motor rehabilitation after brain injuries, depression, and chronic pain. Recently, it has been explored as a potential technique to modulate pain and increase muscle strength in peripheral musculoskeletal pathologies [31].

In the study by Jamebozorgi et al. [34], the experimental group was in a laboratory without external stimuli, whereas the control group was not, which may have influenced the results. However, more studies need to be carried out in the future with similar experimental conditions and with a longer follow-up period, such as the study by Rush et al. [41], which measures the effectiveness of transcranial electrical stimulation after only one session, and in a larger population, as only 43 individuals were included in these two studies.

Given the authors’ conclusions and the complexity and cost of implementing the technique, it is currently difficult to recommend the use of tDCS in rehabilitation protocols following AMI. Moreover, integrating tDCS into rehabilitation protocols requires careful consideration of stimulation parameters and patient selection to ensure safety and efficacy.

#### 4.2.4. High-Tone Therapy

High-tone therapy is a form of electrotherapy that utilizes high-frequency oscillations to stimulate cellular activity and promote tissue healing. The mechanism of this intervention involves modulating both the frequency and amplitude to induce cellular resonance, thereby enhancing metabolic processes and facilitating the repair of nerve, muscle, and bone tissues. This dynamic stimulation is believed to improve microcirculation, enhance cell function, and promote tissue repair, leading to reduced chronic pain and effective long-term pain relief [35].

The results regarding this approach should be interpreted with caution because the experimental group had a much higher weekly workload (1 h of therapeutic exercises combined with 1 h of quadriceps stimulation by high-tone therapy) than the control group [37]. Once again, it can be questioned whether these results are due to the use of high-tone therapy or to the increase in quadriceps stimulation, which was twice as high in the experimental group as in the control group. Once again, these data are limited to a single study, and further research is needed to ensure that implementing high-tone therapy can be beneficial in managing chronic pain conditions and enhancing tissue repair.

### 4.3. Other Interventions

#### 4.3.1. Cryotherapy

Cryotherapy involves the application of cold to the body to reduce the temperature of adjacent tissues, aiming to alleviate pain, decrease inflammation, and promote recovery [22,37].

Our results showed that the use of cryotherapy before therapeutic exercise led to an increase in strength gain. However, it was no better than the comparison groups. The effect of cryotherapy may be justified because cryotherapy may increase motor plate excitability and afferent messages from the periphery, which would increase cortical responsiveness and facilitate strength gains during exercises performed immediately after cryotherapy [30,31]. Further studies are needed to evaluate if cryotherapy adds clinical value to the intervention and evaluate if cryotherapy has any adverse effects, as several studies have shown that cryotherapy can have detrimental effects on the activity of macrophages, leukocytes, satellite cells, and myogenesis [68,69,70,71]. It is known that these components play a fundamental role in the inflammatory process and therefore in tissue repair. So, from a clinical perspective, further studies are needed to verify if applying cryotherapy immediately after injury or surgery can minimize swelling and pain, allowing for earlier initiation of rehabilitation activities.

#### 4.3.2. Motor Imagery

Motor imagery is a cognitive process where an individual mentally simulates a specific action without actual physical execution. Studies have widely demonstrated that thinking about a specific movement (motor gesture) activates the cortical areas corresponding to the movement in the same way as performing the motor action, facilitating the rehabilitation of motor action, even in the context of musculoskeletal injuries [25,39]. Regarding our results, although the use of motor imagery is described by the authors of the included study as an intervention suitable for the acute phase, when the decrease in activity of the motor areas is greater, a beneficial effect can be observed here even in the final phase of rehabilitation, since this study was carried out on patients who had already returned to practice [23]. The simple visualization of a movement can activate the corresponding motor areas, making it an inexpensive and easy-to-implement therapy. For the clinician, incorporating motor imagery into rehabilitation can be particularly useful when physical practice is limited due to pain or immobilization, enhancing motor learning and recovery. Future studies could confirm the interest of motor imagery in the acute and chronic phases, as this therapy seems promising in reducing the cortical inhibition present after ACL injury or reconstruction.

#### 4.3.3. KT

KT involves the application of an elastic adhesive tape, aiming to improve range and flexibility, reduce pain, improve proprioception, strength, stability, lymphatic return, increase blood flow, or prevent injuries [72].

Our results suggest that KT can be a viable, safe, and inexpensive solution in the immediate postoperative period to reduce edema and control pain. The tape is thought to have a mechanical effect, exerting superficial traction on the skin [73] and providing a proprioceptive effect with resistance when stretched. The application of a fake tape shows significantly lower results compared to the application of a real tape [22]. The same conclusion can be found in other musculoskeletal conditions where the use of tape is compared with a fake tape, confirming that the placebo effect is not effective, or at least not as effective, as the use of real tape [74,75,76].

#### 4.3.4. Vibration

Local vibration or whole-body vibration therapy is a neuromuscular therapy that induces oscillations producing rapid and short-duration changes in the length of the musculo-tendinous complex [43]. Vibrations can increase the excitability of motoneurons, synchronization of motor units, plasma concentration, testosterone, or growth hormone, which would improve proprioceptive feedback, balance, muscle trophism, or muscle strength when combined with exercises [24,43].

The cost–benefit ratio of vibration therapy, the inconsistencies between studies, and the potential adverse effects mentioned in the studies (increased pain during exercise and joint edema) do not justify the use of vibration therapy in rehabilitation protocols to date.

### 4.4. Limitations

Although only randomized controlled trials were included, as required by the systematic review methodology, there is always the possibility of some publication bias. The results of this review are based on the findings and conclusions of the authors of the different articles included, often depending on the search criteria, bibliographic sources, and inclusion and exclusion criteria. The exclusion of patients with concomitant injuries or postoperative complications led to a reduction in the number of randomized controlled trials that met the inclusion criteria and were therefore eligible for inclusion. Some newer interventions to modify cortical excitability could not be included because of the current lack of robust evidence on this topic.

One of the potential limitations of this review is that in all but one of the included studies [31], the populations included were patients who had suffered an ACL injury with or without reconstruction, without prior assessment of whether they had had an AMI. With a prevalence of just over one in two patients, it can be said that the results should be highly nuanced, as it is different to rehabilitate a patient without an AMI and a patient with a grade I or III AMI. For this reason, we included the presence of AMI indicators as eligibility criteria. However, we suggest that in the future, high-level evidence studies should be conducted in a population with a diagnosis of AMI through a scientifically validated classification.

There is also a recurrent power bias in the randomized trials included in this review that is a difference in volume or intensity between the control group, which received a standard rehabilitation protocol, and the experimental group. When quantifying the mechanical load between the two groups, there is a difference, sometimes significant. This makes it difficult to interpret the results, because the significant difference found may only be due to a higher volume of work.

### 4.5. General Considerations

The clinical significance of these findings is evident in the observed improvements in functional mobility and pain reduction among patients undergoing specific physiotherapy interventions. Implementing these interventions in clinical practice requires the consideration of factors such as resource availability, clinician expertise, and patient adherence and preferences. To facilitate the integration of our research into routine patient care, we suggest that clinicians carry out the following:

- Perform a comprehensive patient assessment to determine the extent of AMI, considering factors such as muscle strength deficits, joint range of motion limitations, functional impairments, comorbidities, and rehabilitation goals. By achieving this, the clinician will be able to define an individualized intervention.
- Employ a combination of interventions; this multimodal approach can lead to synergistic effects, enhancing overall rehabilitation outcomes.
- Provide patients with educational resources explaining AMI, its impact on recovery, and the importance of adherence to prescribed interventions, thus enhancing patient engagement and commitment to the rehabilitation process.
- Establish systems to monitor patient outcomes, allowing for the assessment of intervention effectiveness and identification of areas for improvement.
- Promote the integration of technology, incorporating telehealth platforms to offer remote monitoring and guidance, increasing accessibility for patients unable to attend in-person sessions.
- Encourage collaboration among physiotherapists, physicians, and other healthcare professionals to develop comprehensive rehabilitation plans addressing both the physical and psychological aspects of AMI.

## 5. Conclusions

Given the heterogeneity of physiotherapy interventions described in the literature, the results of this systematic review allow us to conclude that structured exercise should be the first-line intervention. Still, it is necessary to combine it with other interventions to enhance rehabilitation. These findings support the hypothesis that multimodal rehabilitation strategies targeting peripheral and central mechanisms may be more effective in addressing AMI.

These conclusions are based on studies with low-to-moderate risk of bias and good methodological quality, reinforcing the robustness of these findings.

## Figures and Tables

**Table 1:** Main characteristics and results of the included studies.

Study ID	Participants			Intervention	Outcome Measures		Results *				Methodological Quality (PEDro)
	n	Mean Age (y)	Sex (M; F)		Outcome	Instrument	Control Group (CG)	Intervention Group 1 (IG1)	Intervention Group 2 (IG2)	Statistically Significant Results	
Balki et al. 2016 [22]	30	28	30; 0	**CG:** rehabilitation + placebo KT **IG:** rehabilitation + KT	Knee extension limitation Pain Quadriceps MIVC	Goniometer VAS Dynamometer	5th: 1.33 ± 2.96° 10th: 0.33 ± 1.29° - 5th: 5.46 ± 2.79 (Kg-F) 10th: 8.26 ± 2.93 (Kg-F)	5th: 2 ± 2.53° 10th: 0.66 ± 1.75° - 5th: 6.40 ± 229 (Kg-F) 10th: 9.73 ± 3.08 (Kg-F)	-	- - *p* < 0.05 (IG < CG) - -	8 *
Bodkin et al. 2021 [23]	10	26	2; 8	**CG:** sham (passive motion) **IG:** visuomotor therapy	AMT	TMS	100%: +0.001 ± 0.002% 150%: −0.004 ± 0.1%	100%: +0.58 ± 1.3% 150%: +4.77 ± 3.1%	-	*p* < 0.03 (IG > CG) *p* < 0.004 (IG > CG)	7
Christanell et al. 2012 [24]	16	30	12; 4	**CG:** standard rehabilitation **IG:** standard rehabilitation +EMG biofeedback (BFB)	Passive knee extension ROM Passive knee extension deficit Muscle activation of VMO	High Heel distance test Goniometer EMG	17.4 ± 15.3mm −1.2° extension 70.3% ± 45.8%	2.3 ± 3.2mm + 5.1° extension 124.9% ±52%	-	*p* < 0.01 (IG > CG) *p* < 0.02 (IG > CG) *p* < 0.002 (IG > CG)	7
da Costa et al. 2019 [25]	44	27	44; 0	**CG:** protocoled exercise in a turned-off vibrating platform **IG:** protocoled exercise in a turned-on vibrating platform	Knee extension total work Quadriceps activity	Isokinetic dynamometer: 60°/s EMG	819.10 ± 198.8 Joules 215.4 ± 58.18%	671.57 ± 281.7 Joules 176.8 ± 78.0%	-	-	6 *
Draper and Ballard 1991 [26]	30	30	16; 14	**CG:** exercise + NMES **IG:** exercise + EMG BFB	Knee extension isometric peak torque Knee extension ROM	Isokinetic dynamometer Goniometer	+37.9 ± 12.4% 0.6 ± 2.5°	+46.4 ± 10.5% 0.0 ± 0.0°	-	*p* = 0.04 (IG > CG) -	6
Feil et al. 2011 [27]	96	32	22; 74	**CG:** exercise **IG1:** exercise + NMES Polystim **IG2:** exercise + NMES Kneehab	Knee extensors isokinetic strength Knee function Knee joint laxity	Isokinetic dynamometer: 180°/s Isokinetic dynamometer: 90°/s Shuttle run Single-legged hop Lysholm score Tegner score KT1000 Arthrometer difference	180°: 0.05 ± 0.31 Nm/kg 90°: 0.11 ± 035 Nm/kg −1.03 ± 1.49 s 17.59 ± 29.51 cm 98.31 ± 2.82 4.84 ± 0.85 0.38 ± 0.49 mm	180°: 0.07 ± 027 90°: 0.094 ± 0.40 −0.66 ± 1.27 s 20.63 ± 26.17 cm 97.45 ± 3.20 4.86 ± 0.60 0.38 ± 0.56 mm	180°: 0.37 ± 0.53 90°: 0.49 ± 0.68 −2.06 ± 3.06 s 38.57 ± 40.67 cm 99.06 ± 2.19 5.76 ± 1.42 0.36 ± 0.49 mm	*p* < 0.001 (IG2 > CG) *p* < 0.001 (IG2 > CG) *p* < 0.001 (IG2 > IG1 AND IG2 > CG) *p* < 0.001 (IG2 > IG1 AND IG2 > CG) *p* < 0.001 (IG2 > IG1 AND IG2 > CG) - -	8 *
Fu et al. 2013 [28]	48	24	32; 16	**CG:** rehabilitation **IG:** rehabilitation + WBV	Knee extensors peak torque Proprioception (JPS) Postural control Knee function Knee joint laxity	Biodex dynamometer 60°/s Biodex dynamometer 180°/s Biodex dynamometer Biodex stability system Single-legged test Triple hop test Shuttle run test Carioca test KT1000 Arthrometer difference	60°/s:120.0 ± 34.9 N.m 180°/s: 90.0 ± 22.2 N.m 477 ± 2.93° 5.57 ± 1.53 129.5 ± 38.4 cm 365.2 ± 49.4 cm 9.5 ± 1.7 s 14.0 ± 3.0 s 1.4 ± 1.2 mm	60°/s: 139.7 ± 32.4 N.m 180°/s: 98.9 ± 224 N.m 407 ± 2.17° 520 ± 230 140.8 ± 27.1 cm 381.2 ± 59.5 cm 9.1 ± 0.8 s 13.7 ± 2.7 s 1.4 ± 1.3 mm	-	- - - *p* = 0.013 (IG > CG) *p* = 0.022 (IG > CG) - *p* = 0.036 (CG > IG) - -	8 *
Harput et al. 2019 [29]	48	29	-	**CG:** ACL rehabilitation **IG1:** ACL rehabilitation + concentric cross-exercise **IG2:** ACL rehabilitation + excentric cross-exercise	Quadriceps MIVC Knee function	Isokinetic dynamometer: 60°/s IKDC One-leg hop for distance test (OLHDT)	12 weeks: 2.2 ± 0.5 24 weeks: 2.4 ± 0.3 IKDC: 79.4 ± 9.3 OLHDT: 91.9 ± 9.1	12 weeks: 2.5 ± 0.5 24 weeks: 2.9 ± 0.4 IKDC: 82.1 ± 9.6 OLHDT: 88.9 ± 10.5	12 weeks: 2.5 ± 0.4 24 weeks: 3.0 ± 0.5 IKDC: 84.1 ± 9.4 OLHDT: 91.8 ± 13.0	*p* < 0.001 (IG1 > CG AND IG2 > CG) *p* < 0.01 (IG1 > CG AND IG2 > CG) - -	7 *
Hart et al. 2012 [30]	30	32	20; 10	**CG:** exercise **IG1:** exercise + cryotherapy **IG2:** exercise + TENS	Quadriceps function: . MIVC (side–side ratio) . CAR Knee function Pain Knee extension ROM Edema	Dynamometer IKDC VAS - Knee girth measurement	1.10 ± 1.0 83.2 ± 13.8% 57.0 ± 8.7 1.6 ± 0.9 −0.7 ± 1.2 1.3 ± 3.2	0.82 ± 0.14 77.7 ± 14.0% 58.3 ± 17.8 2.2 ± 1.2 −0.4 ± 2.9 0.5 ± 0.7	0.95 ± 0.15 78.5 ± 12.1% 59.6 ± 13.6 2.1 ± 2.1 0.8 ± 2.1 −0.1 ± 0.5	CG, IG1 and IG2: *p* < 0.05 (pre vs. post) CG, IG1 and IG2: *p* < 0.05 (pre vs. post) CG, IG1 and IG2: *p* < 0.05 (pre vs. post) CG, IG1 and IG2: *p* < 0.05 (pre vs. post) - -	7 *
Hart et al. 2014 [31]	30	27	10; 20	**CG:** exercise **IG1:** cryotherapy **IG2:** exercise + cryotherapy	Quadriceps function: . MIVC (side–side ratio) . CAR	Dynamometer	1.6 ± 0.7 (Nm/kg) 83.4 ± 8.4%	1.7 ± 0.4 (Nm/kg) 80.4 ± 10.5%	2.2 ± 0.7 (Nm/kg) 88.2 ± 5.5%	IG2: *p* < 0.002 (pre vs. post) -	7 *
Hughes et al. 2019 [32]	24	29	17; 7	**CG:** HL-RT 70% 1RM **IG:** BFR-RT 30% 1RM	Extensor muscles strength Pain Knee extension ROM Physical function Effusion Knee joint laxity	Unilateral 10RM Isokinetic dynamometer: . 60°/s . 150°/s . 300°/s KOOS (pain scale) ** Goniometer IKDC score SEBT Flexible tape measure KT1000 Arthrometer difference	106 ± 21% (injured limb) . 60°/s: decreased peak torque . 150°/s: decreased peak torque . 300°/s: decreased peak torque 22.00 ± 7.48 −0,08 ± 0,67 23.33 ± 8.76 increased in the 3 directions −1.0 ± 0.7 1.3 ±0.8 mm	104 ± 18% (injured limb) . 60°/s: decreased peak torque . 150°/s: - . 300°/s: - 39.75 ± 11.74 0.00 ± 0.85 35.63 ± 7.06 increased in the 3 directions −2.3 ± 0.9 1.1 ± 1.7 mm	-	- - CG: *p* < 0,05 CG: *p* < 0.05 *p* < 0.05 (IG > CG) - *p* < 0.01 (IG > CG) *p* < 0.05 (IG > CG) *p* < 0.01 (IG > CG) CG and IG: *p* < 0.05 (pre vs. post)	7 *
Iversen et al. 2016 [33]	24	CG: 30 IG: 25	14; 10	**CG:** exercise **IG:** exercise + BFR	Quadriceps ACSA	Magnetic resonance	−13.1 ± 1.0%	−13.8 ± 1.1%	-	CG and IG: *p* < 0.0001 (pre vs. post)	7 *
Jamebozorgi et al. 2022 [34]	33	21	33; 0	**CG:** exercise **IG1:** exercise + BFB **IG2:** exercise + tDCS	Proprioception Functional balance	Knee absolute error: . 30° . 45° . 90° SEBT: . Anterior (A) . Anterior lateral (AL) . Lateral (L) . Lateral posterior (LP) . Posterior (P) . Posterior medial (PM) . Medial (Med) . Anterior medial (AM)	. 30°: 2.22 ± 1.23 . 45°: 5.20 ± 3.03 . 90°: 7.60 ± 0.79 . A: 1.5 ± 0.83 . AL: 2.8 ± 1.27 . L: 6.8 ± 2.47 . LP: 3.2 ± 3.53 . P: 5.7 ± 2.02 . PM: 12.13 ± 3.73 . Med: 4.9 ± 0.11 . AM: 4.47 ± 2.47	. 30°: 7.82 ± 6.49 . 45°: 11.41 ± 4.93 . 90°: 7.52 ± 5.46 . A: 11.80 ± 2.94 . AL: 12.20 ± 10.33 . L: 7.70 ± 4.90 . LP: 13.70 ± 3.78 . P: 8.10 ± 2.73 . PM: 10.39 ± 6.21 . Med: 14.00 ± 1.18 . AM: 11.82 ± 1.86	. 30°: 4.00 ± 1.83 . 45°: 7.52 ± 3.79 . 90°: 1.60 ± 2.72 . A: 12.40 ± 0.23 . AL: 8.40 ± 6.60 . L: 6.80 ± 6.31 . LP: 12.83 ± 2.33 . P: 10.06 ± 3.60 . PM: 12.22 ± 6.37 . Med: 14.29 ± 4.89 . AM: 11.51 ± 7.25	CG (pre vs. post): *p* < 0.05 (30°, 90°) IG1 (pre vs. post): *p* < 0.05 (30°, 45°, 90°) IG2 (pre vs. post): *p* < 0.05 (30°, 45°, 90°) CG (pre vs. post): *p* < 0.05 (P, PM, Med) IG1 (pre vs. post): *p* < 0.05 (all directions) IG2 (pre vs. post): *p* < 0.05 (A, L, LP, PM, Med, AM)	7 *
Labanca et al. 2022 [35]	34	34	20; 14	**CG:** standard physical therapy **IG:** standard physical therapy + NMES	Quadriceps MIVC	Dynamometer: 30° Dynamometer: 90°	30°: 0.52 ± 0.38 Nm/kg 90°: 0.72 ± 0.22 Nm/kg	30°: 0.85 ± 0.36 Nm/kg 90°: 0.85 ± 0.23 Nm/kg	-	30°: *p* < 0.05 (IG > CG)	5 *
Labianca et al. 2022 [36]	52	29	52; 0	**CG:** standard rehabilitation **IG:** standard rehabilitation + KT	Pain Edema Passive extension ROM Knee function	VAS Knee girth measurement Goniometer KOOS Tegner–Lysholm Knee Scale	2.2 ± 1.3 −2.75 ± 1.4% 91.5 ± 4.5° 61.9 ± 5.3 76.00 ± 5.50	1.9 ± 0.9 −7.6 ± 2.9% 101.0 ± 9.6° 67.3 ± 5.9 85.0 ± 9.3	-	- *p* = 0.006 (IG > CG) - - -	8 *
Ogrodzka-Ciechanowicz et al. 2021 [37]	36	30	35; 0	**CG:** rehabilitation **IG:** rehabilitation + high-tone therapy	Knee extensors strength Knee extension ROM Knee function Pain	Dynamometer: 90° Goniometer Lysholm scale VAS	20.19 ± 0.6 (N/kg) 3.00 ± 0.99° 85.00 ± 8.71 pts 3.00 ± 0.8	23.28 ± 0.7 (N/kg) 0.0 ± 0.15° 94.00 ± 7.01 pts 2.00 ± 0.4	-	*p* = 0.028 (IG > CG) *p* = 0.048 (IG > CG) *p* = 0.035 (IG > CG) -	7 *
Oliveira et al. 2016 [38]	47	29	47; 0	**CG:** no intervention **IG1:** placebo **IG2:** KT	Quadriceps muscle performance: . Concentric peak torque . Eccentric peak torque . Concentric muscle activation . Eccentric muscle activation Postural balance: . Amp A/P . Amp L/L	Isokinetic dynamometer: 60°/s EMG Baropodometry	197.3 ± 52.2% 267.3 ± 59.8% 97.5 ± 51.4% 103.9 ± 16.2% 16.0 ± 3.5 mm 8.2 ± 2.9 mm	195.0 ± 85.2% 263.3 ± 79.1% 119.4 ± 25.5% 103.0 ± 20.8% 14.9 ± 5.5 mm 9.0 ± 3.3 mm	160.6 ± 61.3% 208.3 ± 92.1% 116.8 ± 24.7% 104.1 ± 24.1% 13.6 ± 6.4 mm 7.2 ± 3.0 mm	- - - - - ?	5
Pamukoff et al. 2016 [39]	20	21	4; 16	**CG:** isometric squat **IG1:** isometric squat + WBV **IG2:** isometric squat + LMV	Hoffmann reflex Quadriceps isometric peak torque CAR	EMG Isokinetic dynamometer: 60°	increased decreased decreased	increased increased increased	increased increased increased	- *p* < 0.05 (IG1 and IG2 > CG) *p* < 0.001 (IG1 and IG2 > CG)	7 *
Papandreou et al. 2012 [40]	42	24	42; 0	**CG:** exercise **IG1:** exercise + 3x/week cross-eccentric exercise **IG2:** exercise + 5x/week cross-eccentric exercise	MIVC	Isokinetic dynamometer: 60°/s	−37.83 ± 16.90%	−16.25 ± 24.70%	−6.30 ± 26.01%	*p* < 0.05 (IG1 and IG2 > CG)	6 *
Rush et al. 2020 [41]	10	23	5; 5	**CG:** sham **IG:** tDCS (crossover study)	Muscle function .MIVC . %EMGmax Knee function	Isokinetic dynamometer: 90° EMG KOOS **	VM: −18.9% and VL: −25.9% −10.1% + 3.1%	VM: −12.1% and VL: −14.8% −8.9% +4.7%	-	CG and IG: *p* < 0.05 (pre vs. ost) CG and IG: *p* < 0.05 (pre vs. post) CG and IG: *p* < 0.05 (pre vs. post)	8

A = anterior; ACSA = anatomical cross-section area; AL = antero-lateral; AM = anterior medial; Ampl A/P = displacement amplitude antero-posterior; Ampl L/L = displacement amplitude latero-lateral; AMT = active motor threshold; BFB = biofeedback; BFR-RT = blood flow restriction resistance training; CAR = central activation ratio; CG = control group; CMJ = countermovement jump; EMG = electromyography; F = female; HL-RT = heavy-load resistance training; IG = intervention group; IKDC = International Knee Documentation Committee; JPS = joint position sense; KOOS = knee injury and osteoarthritis outcome score; KT = Kinesio tape; L = lateral; LMV = local muscle vibration; LP = lateral posterior M = male; Med = medial; MIVC = maximal isometric voluntary contraction; NMES = neuromuscular electrical stimulation; OLHDT = one-leg hop for distance test; P = posterior; PM = postero-medial; ROM = range of motion; STSTS = sit-to-stand-to-sit; SWM = Semmes–Weinstein manofilament; tDCS = transcranial direct-current stimulation; TMS = transcranial magnetic stimulation; VAS = visual analog pain scale; VMO = vastus medialis oblique; WBV= whole-body vibration; Y= years; ? = conflicting results between the table results (*p* = 0.08) and the Results Section, where the authors describe that no significant differences were found in the three groups, nor between the three groups. * The results presented (mean, standard deviations, *p* values) are solely the last evaluation performed. ** As 0 represents extreme pain and 100 represents no pain, an increase in pain score is indicative of a reduction in pain.

**Table 2:** Evaluation of the methodological quality of the included studies (PEDro Scale).

Studies	Eligibility Criteria and Source	Random Allocation	Concealed Allocation	Baseline Comparability	Blinding of Participants	Blinding of Therapists	Blinding of Assessors	Adequate Follow-Up (>85%)	Intention-To-Treat Analysis	Between-Group StatisticalComparisons	Reporting of Point Measures and Measures of Variability	Total Score	Qualitative Result
Balki et al. 2016 [22]	1	1	1	1	1	0	0	1	1	1	1	8 *	Good
Bodkin et al. 2021 [23]	1	1	1	0	1	0	0	1	1	1	1	7	Good
Christanell et al. 2012 [24]	1	1	1	1	0	0	0	1	1	1	1	7	Good
da Costa et al. 2019 [25]	1	1	1	1	0	0	0	1	0	1	1	6 *	Good
Draper and Ballard 1991 [26]	1	1	0	1	0	0	0	1	1	1	1	6	Good
Feil et al. 2011 [27]	1	1	1	1	0	1	0	1	1	1	1	8 *	Good
Fu et al. 2013 [28]	1	1	1	1	0	1	0	1	1	1	1	8 *	Good
Harput et al. 2019 [29]	1	1	0	1	0	0	1	1	1	1	1	7 *	Good
Hart et al. 2012 [30]	1	1	1	1	0	0	0	1	1	1	1	7 *	Good
Hart et al. 2014 [31]	1	1	0	1	0	0	1	1	1	1	1	7 *	Good
Hughes et al. 2019 [32]	1	1	1	1	0	0	0	1	1	1	1	7 *	Good
Iversen et al. 2016 [33]	1	1	1	1	0	0	0	1	1	1	1	7 *	Good
Jamebozorgi et al. 2022 [34]	1	1	1	1	0	0	0	1	1	1	1	7 *	Good
Labanca et al. 2022 [35]	1	1	0	0	0	0	0	1	1	1	1	5 *	Fair
Labianca et al. 2022 [36]	1	1	1	1	0	1	0	1	1	1	1	8 *	Good
Ogrodzka-Ciechanowicz et al. 2021 [37]	1	1	1	1	0	0	0	1	1	1	1	7 *	Good
Oliveira et al. 2016 [38]	1	1	0	0	0	0	0	1	1	1	1	5	Fair
Pamukoff et al. 2016 [39]	1	1	0	1	0	0	1	1	1	1	1	7 *	Good
Papandreou et al. 2012 [40]	1	1	0	1	0	0	0	1	1	1	1	6 *	Good
Rush et al. 2020 [41]	1	1	1	1	1	0	0	1	1	1	1	8	Good

* Studies that evaluate complex interventions (e.g., exercise). For these, a total PEDro score of 8/10 is considered optimal.

## Appendix A

**Table A1:** Specific search strategies used in the different databases and their results.

**Database**	**Last Search Date**	
Pubmed	7 February 2023	
**Search Expression**	**Filters Applied**	**Results**
(quadriceps inhibition) OR (arthrogenic muscle inhibition) OR (quadriceps weakness) AND (ACL) OR (ACLR) AND (Physiotherapy intervention) OR (physical therapy intervention) OR (intervention) NO (Arthrofibrosis) OR (bilateral impairment)	English, adults +18 years old	1117
**Database**	**Last Search Date**	
Google Scholar	2 March 2023	
**Search Expression**	**Filters Applied**	**Results**
All following words: Quadriceps inhibition, arthrogenic muscle inhibition, quadriceps weakness, ACL intervention Exact expression: Arthrogenic muscle inhibition None of these words: Arthrofibrosis bilateral impairment		1318
**Database**	**Last Search Date**	
Cochrane Library	1 October 2023	
**Search Expression**	**Filters Applied**	**Results**
Arthrogenic muscle inhibition	Title, abstract, keyword	32
**Database**	**Last Search Date**	
EMBASE	1 October 2023	
**Search Expression**	**Filters Applied**	**Results**
Quadriceps inhibition		175
